# Chronic Lymphocytic Leukemia: Prognostic Factors in the Era of Novel Drugs

**DOI:** 10.3390/cancers16152732

**Published:** 2024-07-31

**Authors:** Antonio Urso, Enrica Antonia Martino, Antonio Cuneo, Massimo Gentile, Gian Matteo Rigolin

**Affiliations:** 1 Hematology Unit, St Anna University Hospital, 44124 Ferrara, Italy; antonio.urso@unife.it (A.U.); cut@unife.it (A.C.); 2 Hematology Unit, Azienda Ospedaliera Annunziata, 87100 Cosenza, Italy; enricaantoniamartino@gmail.com (E.A.M.); massim.gentile@tiscali.it (M.G.); 3 Department of Pharmacy, Health and Nutritional Science, University of Calabria, 87036 Rende, Italy

**Keywords:** chronic lymphocytic leukemia, BTK inhibitors, BCL-2 inhibitor, prognostic scores, *TP53* abnormalities, *IGHV*, complex karyotype

## Abstract

**Simple Summary:**

The treatment of chronic lymphocytic leukemia (CLL) has dramatically changed following the availability of new drugs with targeted mechanisms of action. Traditional and new prognostic factors are being investigated to individualize and guide these new treatments. In this review, we discuss the clinical relevance of genomic factors including immunoglobulin heavy chain variable (*IGHV*) mutational status, *TP53* abnormalities, complex karyotype, and prognostic scores in relation to new targeted agents.

**Abstract:**

Novel drugs have profoundly changed the outcomes in chronic lymphocytic leukemia (CLL) patients, and the traditional prognostic factors that were identified in the era of chemoimmunotherapy need to be validated in the context of these new targeted therapies. Currently, the most important prognostic genetic biomarkers are the immunoglobulin heavy chain variable (*IGHV*) mutational status, genetic aberrations including del(17p)/*TP53* abnormalities, and the complex karyotype. In this review, we discuss the prognostic role of these genomic markers in relation to novel treatments. Moreover, we present and discuss new scoring systems that were elaborated and validated in the era of new drugs. In routine clinical practice, the application of an extensive genomic work-up with validated prognostic markers could improve the identification of “very high-risk” CLL patients who could benefit from novel, more effective targeted treatments.

## 1. Introduction

Chronic lymphocytic leukemia (CLL) is a low-grade lymphoproliferative neoplasm characterized by the accumulation of clonal B-cells expressing CD5, CD19, CD20(dim), and CD23 in the lymphoid organs and blood [1]. CLL represents the most prevalent type of hematological neoplasm in Western countries, and it is characterized by a relative clinical heterogeneity, varying from an indolent to an aggressive course with shorter progression-free survival (PFS) and overall survival (OS) [2]. This clinical variability reflects CLL’s biological and molecular diversity. Thus, there has been a need to develop tools aiding clinicians in better prognostic assessment. The Rai and Binet systems, pivotal in CLL’s prognostic history, remain standard staging systems. Both systems assess lymphocytosis, lymphadenopathy, hepatomegaly, or splenomegaly, and anemia or thrombocytopenia. The Rai system classifies patients into low-, intermediate-, and high-risk subgroups, each associated with distinct survival outcomes. These range from a median OS exceeding 10 years for low-risk cases to a median OS as short as 1.5 years for high-risk patients [3]. In contrast, the Binet system categorizes patients into three subgroups (A, B, and C), with group C having the worst prognosis. Both staging systems allow for the stratification of CLL patients into three risk groups (low, intermediate, and high), each of which are characterized by different survival (150, 101, and 19 months, respectively) [4].

In the era of chemoimmunotherapy, the advent of prognostic biomarkers has made the Rai and Binet staging systems overly simplistic for stratifying CLL patients. Currently, the crucial prognostic biomarkers routinely employed in clinical practice include the immunoglobulin heavy chain variable (*IGHV*) mutational status, cytogenetic aberrations such as the deletion of 17p (del(17p) and 11q (del11q), complex karyotype, and mutations of the *TP53*, *NOTCH1*, *ATM*, and *BIRC3* genes [5,6,7,8,9,10,11,12]. 

The unmutated status of *IGHV* is associated with a higher rate of disease progression, shorter time to first treatment, and decreased OS. On the contrary, a mutated status is indicative of a more indolent course [5]. 

In the era of chemoimmunotherapy, mutations in *TP53*, a gene encoding for a tumor suppressor protein located on the short arm of chromosome 17, either alone or in combination with del(17p), have been linked to an unfavorable prognosis as demonstrated by a shorter time to next treatment and decreased or lack of response to treatment with Fludarabine–Cyclophosphamide–Rituximab (FCR) and Bendamustine–Rituximab [6,7]. The *ATM* gene, essential for cell cycle checkpoint activation and DNA damage response, is affected in del(11q) and present in 15–20% of CLL cases at diagnosis [8]. Often associated with unmutated IGHV status and bulky disease, del(11q), combined with *TP53* alterations, leads to highly adverse outcomes in CLL patients, promoting a clonal advantage in vitro and in vivo [9]. Treatment-naive CLL patients with del(11q) can achieve sustained remission with FCR [10]. Additionally, a complex karyotype is defined as the occurrence of three or more chromosomal abnormalities in one clone [11], while high cytogenetic complexity is characterized by the presence of five or more chromosomal abnormalities [11]. A complex karyotype occurs in up to 15% of patients and is frequently associated with unmutated *IGHV* status. It serves as a stronger predictor than del(17p) of an inferior outcome in relapsed/refractory CLL patients, even in the era of new small molecules [12]. 

Although less common in clinical practice, the detection of mutations in *NOTCH1*, *ATM*, and *BIRC3* genes appears very valuable in assessing CLL patient prognosis in the era of chemoimmunotherapy, as *BIRC3* mutations confer a 10-year survival rate of 29% [13], while *NOTCH1* and *ATM* mutations have been associated with adverse prognostic effects [14]. 

Regarding the significance of biochemical abnormalities as a prognostic indicator, serum β2-microglobulin (β2M) has been routinely considered a feasible biomarker, where elevated levels (>2.0 mg/L) correlate with a worse outcome [15]. Among cell surface markers, zeta chain-associated protein kinase 70 (*ZAP-70*), CD38, and CD49d expression are associated with earlier disease progression, shorter time to first treatment, and a sub optimal response to chemoimmunotherapy [16,17].

The CLL International Prognostic Index (CLL-IPI) was formulated by analyzing clinical, biological, and genetic parameters including age, clinical stage, *TP53* status, *IGHV* status, and serum β2M, utilizing data from patients enrolled in eight randomized clinical trials in 2016. This index facilitated cases’ stratification into four distinct groups, each exhibiting different outcomes, thereby serving as a surrogate predictor of OS and time to first treatment in newly diagnosed CLL patients [18,19]. The drawback of this score lies in its lack of validation in the setting of relapsed/refractory CLL patients. Presently, the CLL treatment armamentarium has drastically improved with the availability of new small molecules in daily clinical practice, facilitating precision medicine. Bruton tyrosine kinase inhibitors (BTKi) and B-cell lymphoma 2 inhibitors (BCL2i) have demonstrated efficacy in improving PFS and OS with manageable adverse effects, even among CLL patients harboring negative biological prognostic factors. Moreover, these therapies have enhanced the depth of response, leading to improvements in treatment-free remission [20]. With the introduction of small molecules in clinical practice, it has become crucial to validate prognostic factors in the context of targeted drugs. Very recently, the German CLL study group reassessed the prognostic relevance of CLL-IPI in the era of targeted therapies, through an exploratory analysis of a pooled set of phase 2 and 3 clinical trials. Overall, the authors found that survival was improved with targeted therapies in comparison to chemoimmunotherapy and that CLL-IPI retained its prognostic role for PFS in the first-line treatment of patients; however, due to the short period of observation, a longer follow-up would be needed to assess the impact on OS. The authors also underscored that there is a need for the continuous evaluation of prognostic factors due to the ongoing evolving landscape of CLL treatment strategies also focusing on individual agents [21].

Additionally, new prognostic markers, such as the detection of measurable residual disease (MRD), could potentially be integrated into routine prognostic scores. Multi-parameter flow cytometry is currently the most used and standardized technique for MRD evaluation in CLL and, since 2018, the iwCLL guidelines have defined the threshold for undetectable MRD at 10^−4^ [1]. However, MRD status may have different clinical relevance, depending on which novel agent is used. Indeed, while undetectable MRD can frequently be achieved and has been associated with improved PFS in patients treated with Bcl-2 inhibitors, alone or in combination with anti-CD20 monoclonal antibody, only around 10% of patients treated with BTK inhibitors may obtain undetectable MRD, even though this does not preclude effective disease control and prolonged survival [22]. A better understanding of MRD dynamics, particularly in relation to new treatment combinations, could help to identify “very-high risk” CLL patients, who may benefit from more efficacious treatments [23].

In this review, we discuss the clinical outcomes of principal clinical trials with novel agents in relation to genomic factors including *IGHV* mutational status, *TP53* abnormalities, and the complex karyotype, and to the new score systems that have been developed accordingly. 

## 2. BTK Inhibitors (Covalent and Non-Covalent)

### 2.1. IGHV Mutational Status

The more aggressive course of patients with an unmutated *IGHV* configuration (U-CLL) [5,24] is accounted for by studies showing a cellular response to the antigenic engagement of B cell receptors, particularly within proliferation centers, in U-CLL and an anergic response to the antigenic stimulation in mutated cases (M-CLL) [25]. 

The prognostic and predictive role of the *IGHV* mutational status was confirmed in several trials using various chemo-immunotherapy regimens [26,27,28,29,30,31]. In patients treated with chemo-immunotherapy, the unmutated status of *IGHV* was associated with a higher rate of disease progression and worse outcomes [5,6,7]. However, in specific subsets of CLL patients, such as those using *IGHV3*-21, the clinical outcome appears to be independent of the *IGHV* mutational status, although these observations were mainly related to the chemo-immunotherapy era [32]. 

The advent of BTK inhibitors has counteracted the prognostic and predictive significance of this biomarker (Table 1). With the longest follow-up among trials with BTK inhibitors, the RESONATE-2 study still confirms the benefit of Ibrutinib in patients with unmutated *IGHV* status over Clhorambucil [33]. Similar results were observed in multiple randomized controlled trials comparing Ibrutinib ± anti CD20 monoclonal antibody against chemoimmunotherapy regimens in the frontline setting of both elderly and young CLL patients [22,34,35,36]. In high-risk U-CLL patients, similar results were reported in randomized controlled trials with second-generation BTKi (Acalabrutinib and Zanubrutinib versus chemo-immunotherapy) [37,38]. In the ELEVATE-TN trial [37,38], after a median follow-up of 74.5 months, in treatment-naive, unfit U-CLL patients, the median PFS was not reached in the Acalabrutinib-containing arm versus 22.2 months in the control arm of Chlorambucil–Obinutuzumab. Zanubrutinib is a BTK inhibitor, displaying higher potency and selectivity than Ibrutinib and Acalabrutinib, and with a more favorable safety profile. In the phase 3 SEQUOIA study, Zanubrutinib was demonstrated to be superior in terms of PFS in treatment-naive patients compared with Bendamustine–Rituximab [39]. Overall, patients with either mutated or unmutated *IGHV* in the experimental arm had significantly better PFS than patients in the chemo-immunotherapy group. Moreover, PFS was not influenced by *IGHV* mutational status in cases treated with BTK inhibitors. The benefit of BTK inhibitors in patients with *IGHV* borderline and/or harboring specific stereotyped BCR subsets (BCR subset #2) is currently under evaluation [40].

Pirtobrutinib is a selective, non-covalent, and reversible BTK inhibitor with activity towards both wild-type and C481-mutant BTK, commonly associated with resistance to covalent BTK inhibitors. In December 2023, the Food and Drug Administration approved Pirtobrutinib for the treatment of relapsed or refractory CLL patients who have received at least two lines of systemic therapy, including a BTK inhibitor and a Bcl-2 inhibitor, based on results from the phase 1/2 BRUIN trial [41,42]. Patients with U-CLL, representing the majority of cases, have an estimated median PFS of 18.7 months.

**Table 1:** Efficacy outcomes based on *IGHV* mutational status with BTK inhibitors.

Trial	Setting	Treatment	Age Median	No. of Patients	U-CLL N (%)	PFS % M-CLL/U-CLL	OS % M-CLL/U-CLL	Ref.
RESONATE-2	TN	Ibr	73	136	58 (43)	At 7 y 68/58	NR	Barr et al. [33]
		Clb	72	133	60 (45)	At 7 y 17/2	NA	
ECOG ACRIN E1912	TN	Ibr + R	58	354	210 (75)	At 5 y 83/75;	At 5 y 97/95	Shanafelt et al. [22]
		FCR	57	175	71/115 (61.7)	At 5 y 68/33	At 5 y 92/84	
Alliance A041202	TN	Ibr	71	182	77/122 (65)	At 2 y 84/79	NA	Woyach et al. [34]
		Ibr + R	71	182	70/115 (61)	AT 2 y 87/71	NA	
		BR	70	183	71/123 (58)	At 2 y 77/56	NA	
FLAIR	TN	Ibr + R	63	386	194 (50)	At 3 y 91.6/87.8	NA	Hillmen et al. [35]
		FCR	62	385	194 (50)	At 3 y 90.5/74.2	NA	
iLLUMINATE	TN	Ibr + Obi	70	113	66/107 (62)	At 4 y 89/67	NA	Moreno et al. [36]
		Clb + Obi	72	116	57/107 (53)	Median NA/15.2 m	NA	
ELEVATE-TN	TN	Aca + Obi	70	179	103(57.5)	At 4 y 89/86	NA	Sharman et al. [37,38]
		Aca	70	179	119 (66.5)	At 4 y 81/77	NA	
		Clb + Obi	71	177	116 (65.5)	At 4 y 62/4	NA	
SEQUOIA	TN	Zan	70	241	125/234 (53.4)	At 2 y 83.4/88	NA	Tam et al. [39]
		BR	70	238	121/131 (52.4)	At 2 y 77.2/62.8	NA	
RESONATE	R/R	Ibr	67	195	98/134 (73)	Median 48.4/49.7 m	NA	Munir et al. [42]
		Ofa	67	196	84/133 (63)	NA	NA	
ELEVATE R/R	R/R	Aca	66	268	220 (82.1)	At 40.9 m 70.4/40.9	NA	Byrd et al. [43]
		Ibr	65	265	237 (89.4)	At 40.9 m 53.6/48.1	NA	
ALPINE	R/R	Zan	67	327	239 (73.1)	At 2 y 76/72	NA	Brown et al. [44]
		Ibr	68	325	239 (73.5)	At 2 y: 74/60	NA	
BRUIN	R/R	Pirto	67	317	168 (84)	Median 19.4 m	At 24 m 73.2	Mato et al. [41,42]

Abbreviations: BTK, Bruton Tyrosine Kinase; PFS, progression-free survival; OS, overall survival; Aca, Acalabrutinib; Zan, Zanubrutinib; Pirto, Pirtobrutinib; BR, Bendamustine–Rituximab; R, rituximab; FCR, Fludarabine–Cyclophosphamide–Rituximab; Ofa: Ofatumomab; Obi, Obinutuzumab; y, years; m, months; M-CLL: *IGHV* Mutated CLL; U-CLL, *IGHV* Unmutated CLL; NR: not reached; NA, not available.

### 2.2. TP53 Abnormalities

CLL patients harboring a *TP53* disruption are those who probably benefited the most from the introduction of BTK inhibitors, given the poor outcome in the era of chemo-immunotherapy. Subgroup analyses of multiple randomized controlled trials comparing BTK inhibitors ± anti CD20 monoclonal antibodies to chemoimmunotherapy may question the predictive role of *TP53* in the front-line treatment of patients with BTK inhibitors (Table 2). In the iLLLUMINATE trial, the Ibrutinib + Obinutuzumab treatment was associated with a significant PFS benefit in comparison to the control arm, with a PFS that was similar in patients with or without *TP53* abnormalities [36]. Similar results were derived from the ALLIANCE trial [34] and in a pooled analysis across four studies (PCYC-1122e, RESONATE-2, iLLUMINATE, and ECOG-ACRIN E1912) [43].

The effectiveness of first-line Ibrutinib was also evaluated in a large series of 747 CLL patients with *TP53* aberrations in a nationwide study, confirming that Ibrutinib is an effective first-line treatment for CLL patients with *TP53* aberrations who are treated at both large academic centers and community practice hospitals [48]. Analogously, Acalabrutinib has demonstrated a survival benefit in high-risk cytogenetic subgroups, such as those with del(17p) and or mutated *TP53*, both in the ELEVATE-TN trial [37,38] and the ELEVATE-RR study, where PFS and OS were comparable across patients harboring high-risk cytogenetics such as del(17p), del(11q), complex karyotype, or advanced disease, regardless of the number of previous treatments [46]. Notably, in the ELEVATE-TN trial, no PFS advantage was observed in the *TP53* mutant subgroup when obinutuzumab was added to acalabrutinib in comparison to acalabrutinib monotherapy [38].

By contrast, when BTK inhibitors are used in relapsed/refractory CLL patients, *TP53* deficiency seems to maintain its negative predictive significance on PFS [45]. Such negative predictive value of *TP53* disruption in relapsed/refractory settings was questioned by Zanubrutinib in a head-to-head randomized controlled trial with Ibrutinib, which showed a longer PFS in comparison to patients with a *TP53* disruption who received Ibrutinib [47].

Finally, recent data suggest that Pirtobrutinib efficacy seems not to be influenced by *TP53* disruption [41,42].

### 2.3. Cytogenetics

The introduction of novel agents in CLL treatment has questioned the predictive role of a complex karyotype. In the era of chemo-immunotherapy, a complex karyotype, defined as the presence of ≥3 chromosomal aberrations, was associated with a shorter time to first treatment, PFS, or OS [49]. Moreover, the presence of at least five cytogenetic abnormalities which defines a high-complex karyotype, is associated with a negative outcome, independently of *TP53* status, while low or intermediate-complex karyotypes (three or four cytogenetic abnormalities) display a prognostic role only in the presence of *TP53* disruption [11,50].

Nevertheless, in patients treated with BTK inhibitors, the complex karyotype should not be automatically considered as an adverse prognostic factor. In the front-line setting of patients treated with Ibrutinib, there are conflicting data. Indeed, while in the ALLIANCE trial, there was no significant impact on the PFS of the complex karyotype [34], while in the phase 2 GIMEMA LLC1114 trial, the complex karyotype was significantly associated with a shorter PFS in the multivariable analysis [51]. In a retrospective analysis including 456 TN and RR CLL patients treated with a single agent, Ibrutinib, or in combination with an anti-CD20 antibody, multivariable analysis showed that increasing karyotypic complexity was an independent predictor of shorter PFS (hazard ratio, 1.07; *p* < 0.0001) and OS (hazard ratio, 1.09; *p* < 0.0001) [12]. Finally, a phase 1/2 multicenter study evaluating Acalabrutinib in treatment-naive patients, with cases containing ≥3 chromosomal aberrations (n = 12), obtained an overall response rate of 100% and, after a median follow-up of 53 months, PFS and OS were superimposable in patients with or without complex karyotype [52].

### 2.4. Scoring Systems

Different prognostic models have been designed in order to better combine clinical and biological parameters (Table 3). A milestone in scoring systems in the era of Ibrutinib was represented by the four-factor prognostic model [53], which included 720 patients treated with the BTK inhibitor within phase II and III trials. Multivariable analysis and machine-learning algorithms identified four parameters, represented by *TP53* aberration, prior treatment, β2M > 5 mg/L, and LDH > 250 U/L, which permitted the classification of patients into three different groups and that were demonstrated to be independently associated with an inferior PFS and OS. The model also maintained statistical significance for PFS and OS in the treatment-naive cohort. Moreover, the authors investigated the rates of BTK and/or PLCγ2 mutations, which classically confer resistance to Ibrutinib therapy, and found that the cumulative incidence of those mutations was increased in the high-risk group. Given the ease in translating the four-factor prognostic model into clinical practice, this model was also validated in the real-life setting. Indeed, the Italian multicenter working group on CLL (“Campus CLL dataset”) investigated the validity and reproducibility of the four-factor model in terms of PFS and OS, in a cohort of 586 patients treated with Ibrutinib outside of clinical studies as a first-line salvage therapy [54]. In this cohort, the presence of *TP53* aberrations, a relapsed/refractory CLL status, and high LDH or β2-M levels were significantly associated with prognosis both in univariate and multivariate analysis. Moreover, the Italian group devised a survival risk score for Ibrutinib, termed SRSI, to predict OS in the setting of relapsed/refractory CLL patients treated with Ibrutinib outside of clinical trials. Again, the model included simple clinical variables such as LDH, β2-M levels, and hemoglobin, all of which were able to predict the outcome of patients treated with Ibrutinib [55,56].

## 3. BCL-2 Inhibitor (Venetoclax ± Anti CD20 Monoclonal Antibodies)

### 3.1. IGHV Mutational Status

Venetoclax is the only BCL2 inhibitor approved for the treatment of CLL. 

In a pooled analysis of four phase 1 or 2 clinical trials (Table 4) that included relapsed CLL patients mostly treated with Venetoclax monotherapy [59], the *IGHV* mutational status did not correlate with response rates, although U-CLL patients experienced a shorter response duration. Recently, in the VENICE-1 phase 3B trial, in relapsed CLL patients treated with Venetoclax monotherapy, the *IGHV* mutational status did not impact on response rates, whereas U-CLL had a higher rate of undetectable MRD but an inferior PFS [60].

Venetoclax was also investigated in a head-to-head comparison with chemo-immunotherapy, both in frontline and relapsed/refractory settings (Table 4).

In relapsed/refractory CLL, the MURANO study [61] showed that after 5 years of follow-up, Venetoclax–Rituximab was superior to Bendamustine–Rituximab in terms of PFS in all the prespecified subgroups. U-CLL and M-CLL patients treated with Venetoclax–Rituximab exhibited similar response rates, including undetectable MRD at the end of treatment. After 5 years of follow-up, U-CLL patient had a higher rate of MRD conversion with subsequent progressive disease in comparison to M-CLL and a shorter median PFS in the Venetoclax–Rituximab arm [61]. Moreover, in multivariate analysis the mutated *IGHV* status was independently associated with a reduced risk of relapse [61]. The inferior PFS in U-CLL patients treated with Venetoclax–Rituximab could be justified by a faster CLL regrowth in U-CLL cases with undetectable MRD.

In the upfront CLL14 trial, Venetoclax–Obinutuzumab was superior to Chlorambucil–Obinutuzumab in most high-risk subgroups, including U-CLL [62]. PFS was longer in M-CLL than in the U-CLL counterpart in both treatment arms. In multivariate analysis, U-CLL predicted a worse PFS [63]. U-CLL and M-CLL obtained similar rates of undetectable MRD with Venetoclax–Obinutuzumab [62], while the MRD doubling time was not affected by the *IGHV* mutational status only in the Venetoclax–Obinutuzumab arm [64]. In the Venetoclax–Obinutuzumab-treated patients, OS was not influenced by the *IGHV* mutational status [65].

**Table 4:** Efficacy outcomes based on *IGHV* mutational status with Venetoclax.

Trial	Setting	Treatment	Age Median	No. of Patients	No. of Pts with U-CLL (%)	PFS M-CLL/U-CLL	OS% M-CLL/U-CLL	Ref.
CLL14	TN	Ven + Obi	72	216	121 (60.5)	Median: NR/57.3 m	At 5 y 86.6/80.5	Al-Sawaf et al. [62,63]
		Clb + Obi	71	216	123 (59.1)	Median 54.5/26.9 m	At 5 y 87/70.8	
MURANO	R/R	Ven + R	64.5	194	123 (68.3)	Median NR/52.2 m	At 5 y 92.3/80.7	Seymour et al. [61]
		BR	66	195	123 (68.3)	Median 24.2/15.7 m	At 5 y 66.7/61.4	
M12-175, M13-365, M13-982, M14-032	R/R	Ven +/− R	66	436	176 (76)	NA	NA	Roberts et al. [59]
VENICE-1	R/R	Ven	68	258	111 (43)	Median 31.8/28.3 m	NA	Kater et al. [60]

Abbreviations: PFS, progression-free survival; OS, overall survival; NR: not reached; Ven, Venetoclax; Obi, Obinutuzumab; Clb, Chlorambucil; R, Rituximab; BR, Bendamustine-Rituximab; y, years; m, months; M-CLL, *IGHV* Mutated CLL; U-CLL, *IGHV* Unmutated CLL; NA, not available.

### 3.2. TP53 Abnormalities

*TP53* abnormalities (del(17p) and/or *TP53* mutations) have been associated with a reduced PFS in CLL patients treated with Venetoclax as a single agent, but they had no impact on response rates and rate of undetectable MRD [59,60] (Table 5).

The MURANO trial showed that, in comparison to Bendamustine–Rituximab, treatment with Venetoclax–Rituximab was associated to a PFS benefit in patients with del(17p) and/or *TP53* mutations, although, in the Venetoclax–Rituximab arm, a better PFS was still observed in patients without del(17p) and/or *TP53* mutation in comparison to those with *TP53* abnormalities. Moreover, in the Venetoclax–Rituximab arm, the rate of undetectable MRD at the end of treatment seemed to be impaired by the presence of del(17p) as all these patients experienced progressive disease versus 22.2% of patients without del(17p) [61]. Furthermore, *TP53* mutational status was identified as a covariate related to a faster MRD growth rate. Finally, in the Venetoclax–Rituximab arm, patients with del(17p) and/or *TP53* mutation also had a significantly reduced 5-year OS [61]. 

The adverse prognostic significance of *TP53* abnormalities was also confirmed in patients treated up front with a fixed duration Venetoclax–Obinutuzumab regimen. In the CLL14 trial, patients with *TP53* abnormalities had a shorter PFS than those without *TP53* abnormalities. Del(17p) (regardless of *TP53* mutational status) and lymph node size ≥5 cm were the only variables significantly associated to a shorter PFS in multivariable analysis [62,63].

### 3.3. Cytogenetics

The predictive role of genomic complexity was evaluated in CLL patients treated with Venetoclax fixed-duration therapy. In the MURANO trial, high genomic complexity, as defined by the presence of five or more aberrations by array comparative genomic hybridization, was associated to higher rates of MRD positivity at the end of treatment [66] and of undetectable MRD conversion with subsequent progressive disease [61]. Moreover, in the Venetoclax–Rituximab arm, after a median follow-up of 5 years, genomic complexity negatively affected PFS but not OS [61].

In the frontline setting, the CLL14 trial showed that a complex karyotype with ≥3 chromosomal abnormalities maintained its adverse significance in the Chlorambucil–Obinutuzumab arm, whereas in the Venetoclax–Obinutuzumab arm, patients with and without a complex karyotype had similar outcomes for undetectable MRD, PFS, and OS [67].

### 3.4. Scoring Systems

To the best of our knowledge, there are no scoring systems specifically validated for Venetoclax-treated patients; therefore, this is an area of research that could be implemented in future studies. Nonetheless, in the aforementioned BALL score, 389 patients treated in the MURANO trial were selected and included as an external validation dataset [57].

## 4. Combinations of BTK and BCL2 Inhibitors

BTK inhibitors and Venetoclax have a synergistic mechanism of action in that BTK inhibitors have a preferential activity on dividing CLL cells in the lymph nodes, whereas Venetoclax exerts its activity preferentially on resting CLL cells, particularly in peripheral blood [68]. Different clinical trials combining BTK inhibitors and Venetoclax were therefore designed to improve outcome results.

### 4.1. IGHV Mutational Status

In the CAPTIVATE study, after 36 months of median follow-up, in the fixed-duration Ibrutinib–Venetoclax cohort, PFS rates for patients with U-CLL and M-CLL were similar (Table 6). Unexpectedly, U-CLL showed deeper MRD responses than M-CLL, with the best undetectable MRD rates in the bone marrow of 73% for U-CLL and 60% for M-CLL [69]. Of interest, similar results were also obtained in the MRD-guided cohort [70].

In the Ibrutinib–Venetoclax arm of the GLOW study, the 42-month PFS rate was longer in M-CLL than in U-CLL. Nevertheless, in a post hoc analysis, the undetectable MRD rate at the end of treatment was found to be higher and predictive of longer PFS only in U-CLL patients, while the M-CLL cases with detectable or undetectable MRD rates at the end of treatment had similar PFS. Based on these results, the evaluation of MRD seems to be more clinically relevant in U-CLL patients. Finally, in the Ibrutinib-Venetoclax cohort, the *IGHV* mutational status did not impact OS in both univariate and multivariate analysis [71].

In the recently published FLAIR phase 3 trial, after a median follow-up of 43.7 months, the Ibrutinib-Venetoclax arm showed a significant improvement in PFS and OS compared to FCR arm, but only in the U-CLL subgroup. No data are available on the impact of *IGHV* mutational status within the Ibrutinib-Venetoclax arm. Of interest, median time to undetectable MRD was shorter in patients with U-CLL versus those with M-CLL in both the peripheral blood and bone marrow [72]. 

The phase 3 GAIA/CLL13 trial reported a 36-month PFS rate that was slightly shorter in U-CLL treated with Obinutuzumab–Ibrutinib–Venetoclax than in M-CLL patients treated with the same regimen [73].

### 4.2. TP53 Abnormalities

A post hoc analysis of the CAPTIVATE trial showed that patients with *TP53* aberrations (including both del17p and *TP53* mutations) have the same rate of overall and complete responses, and undetectable MRD of patients without *TP53* abnormalities, if treated with Ibrutinib–Venetoclax. With 36 months of follow-up, OS was not impaired by the *TP53* status but, in the PFS analysis, the *TP53* aberrations were associated to a slightly lower PFS rate (81% and 91% for patients with and without *TP53* aberration, respectively) [69,70]. In the GLOW and CLL13 trials, patients with *TP53* aberrations were excluded [71,73].

### 4.3. Cytogenetics

The phase 3 GAIA/CLL13 trial had identified the highly complex karyotype as defined by the presence of five or more karyotypic abnormalities, but not the complex karyotype with three or four chromosomal abnormalities, as an independent adverse prognosticator for PFS [74]. Of interest, the presence of translocations and, particularly, the unbalanced ones was also independently associated with an inferior PFS in the Venetoclax arms as previously observed in single-center series of patients mainly treated with chemo-immunotherapy [75]. CLL 13 data on the complex karyotype are of relevance because they were independent of *TP53* disruptions, a factor frequently associated to complex karyotype [11].

### 4.4. Scoring Systems

To the best of our knowledge, there are no scoring systems specifically validated for combined BTK and BCL2 inhibitor treatments.

## 5. Future Directions: Conclusions

Novel drugs have profoundly changed the outcomes in CLL patients, and it appears evident from clinical trials and from data obtained in patients not included in clinical trials that, nowadays, chemoimmunotherapy has a very limited role in the treatment of CLL patients in both the frontline and relapsed refractory settings.

Targeted therapies have also been shown to improve outcomes in patients with “high-risk” genomic features including the IGHV unmutated status, *TP53* dysregulation, and complex karyotype. However, conflicting data have been observed in clinical trials and real-life observations concerning the possibility of completely overcoming the negative prognostic impact of these high-risk genomic features with novel targeted agents.

The optimal first-line management of CLL patients and the most appropriate sequencing of targeted therapies or their combinations are still a matter of intense research. Actual guidelines and recommendations also suggest the individualization of treatment choices based on the clinical characteristics and preference of the patients. 

In the era of novel targeted therapies, a better definition of prognostic and predictive factors will certainly help us to identify the most appropriate treatment in the different biological and clinical settings. This is of relevance as fixed-duration or continuous treatments are now available, and novel combinations of drugs with different but synergic mechanisms of action are being tested in prospective clinical trials [76].

**Figure 1:**
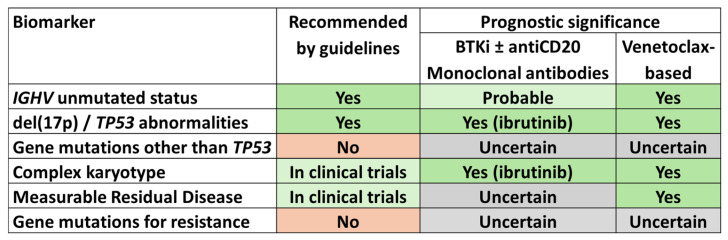
Recommendations and prognostic relevance for genomic biomarkers in CLL patients treated with targeted therapies.

Considering that MRD at the end of induction with a fixed-duration therapy was predictive of longer PFS and clinical benefit in most clinical trials, the European Medicine Agency (EMA) has accepted MRD as the surrogate endpoint for early licensure of new drug awaiting mature PFS data [77]. Because of that, MRD could represent an interesting surrogate clinical endpoint for outcome evaluation, as emerged from ongoing clinical trials (Table 7), and to guide individualized treatment decision, although its clinical utility in the different biological settings requires further research and standardized and reproducible methodologies [78] (Figure 1).

Additionally, mutations involving the *BTK* and *PLCG2* [79,80] or *BCL-2* [81,82] genes are emerging as relevant mechanisms for resistance and progression in patients treated with BTK and BCL2 inhibitors, respectively. The prognostic and predictive significance of these mutations is still a matter of intense research, and it is plausible that, in the future, the study of mutations could guide the choice and sequencing of treatments with novel agents [83].

## Figures and Tables

**Table 2:** Efficacy outcomes based on *TP53* status with BTK inhibitors.

Trial	Setting	Treatment	No. of Pts	Median Age	No. of Pts with *TP53* Abnorms/Total Cases (%)	PFS%	OS%	Ref.
Alliance A041202	TN	Ibr	182	71	15/168 (9)	At 2 y NE	NA	Woyach et al. [34]
		Ibr + R	182	71	20/168 (12)	At 2 y NE	NA	
		BR	183	70	16/174 (9)	At 2 y 7m	NA	
FLAIR	TN	Ibr + R	386	63	2 (1)	NA	NA	Hillmen et al. [35]
		FCR	385	62	1 (<1)	NA	NA	
iLLUMINATE	TN	Ibr + Obi	113	70	13/112 (12)	NE	NA	Moreno et al. [36]
		Clb + Obi	116	72	16/110 (15)	Median 18 m	NA	
ELEVATE-TN	TN	Aca + Obi	179	70	21 (11.7)	At 1 y 95	NA	Sharman et al. [37,38]
		Aca	179	70	19 (10.6)	NA	NA	
		Clb + Obi	177	71	21 (11.9)	At 1 y 19	NA	
NCT01500733	TN	Ibr	34	63	34	At 6 y 61	At 6 y 79	Ahn et al. [44]
SEQUOIA	TN cohort C	Zan	111	70	111/111 (100)	At 2 y 88.9	At 2 y 93.6	Tam et al. [39]
RESONATE	R/R	Ibr	195	67	79/154 (51)	18 m 66	NA	Munir et al. [45]
		Ofa	196	67	68/149 (46)	18 m 0	NA	
ELEVATE R/R	R/R	Aca	268	66	100 (37.3)	NA	NA	Byrd et al. [46]
		Ibr	265	65	112 (42.3)	NA	NA	
ALPINE	R/R	Zan	327	67	30 (9.2)	At 2 y 72.6	NR	Brown et al. [47]
		Ibr	325	68	25 (7.7)	At 2 y: 54.6	NR	
BRUIN	R/R	Pirto	317	67	87/222 (39.2)	Median 16.9 m	NA	Mato et al. [41,42]

Abbreviations: BTK, Bruton Tyrosine Kinase; PFS, progression-free survival; OS, overall survival; NA, not available; NE, not evaluable; NR: not reached; Ibr, Ibrutinib; Clb, Chlorambucil; Aca, Acalabrutinib; Zan, Zanubrutinib; Pirto, Pirtobrutinib; BR, Bendamustine–Rituximab; R, Rituximab; FCR, Fludarabine–Cyclophosphamide–Rituximab; Ofa: Ofatumomab; Obi, Obinutuzumab; y, years; m, months.

**Table 3:** Scoring systems with novel agents in CLL.

Ref.	Score System	CLL Therapy	No. of Patients	Status of Disease	Variables	Categories (Points)	OS Category
Soumerai et al. [57]	BALL score	Ibrutinib Idelalisib Venetoclax	727 897 389	R/R	β2M (≥5 mg/L) LDH > ULN Hb (<110 g/L for women or <120 g/L for men) time to initiation of last therapy (<24 months)	low (0–1) int (2–3) high (4) low (0–1) int (2–3) high (4) low (0–1) int (2–3) high (4)	2y OS 89.7% 79.5% 55.8% 82.6% 61.8% 49.5% 95.1% 84.6% 82.2%
Gentile et al. [55]	SRS_I_	Ibrutinib	541	R/R	Hb (<110 g/L for women or <120 g/L for men) β2M (≥5 mg/L) LDH > ULN	Low (0) Int (1–3) High (4–5)	2y-OS 95.3% 81% 60.6%
Gentile et al. [56]	SRS_i_	R-idelalisib	142	R/R	Hb (<110 g/L for women or <120 g/L for men) β2M (≥5 mg/L) LDH > ULN	Low (0) Int (1–3) High (4–5)	2y-OS 88.6% 69.6% 54.3%
Ahn et al. [53]	4-factor prognostic model	Ibrutinib	720	R/R	*TP53* aberration prior treatment β2M > 5 mg/L LDH > 250 U/L	Low (0–1) Int (2) High (3–4)	3y-OS 93% 83% 63%
Morabito et al. [54]	4-factor prognostic model	Ibrutinib	586	R/R	*TP53* aberration prior treatment β2M > 5 mg/L LDH > 250 U/L	Low (0–1) Int (2) High (3–4)	3y-OS 89.7% 77.8% 60.3%
Molica et al. [58]	CLL-3 model	Ibrutinib	338	R/R	LDH values >UNL Rai stage III/IV early POD	Low (0) Int (1) High (2–3)	3-y OS 91% 84% 65%

Abbreviations: OS, overall survival; R/R. relapsed refractory; POD, progression of disease; UNL, upper normal limit.

**Table 5:** Efficacy outcomes based on *TP53* status with BCL2i.

Trial	Setting	Treatment	Age Median	No. of Pts	No. of Pts with TP53 D (%)	PFS% TP53-ND/D	OS% TP53-ND/D	Ref.
CLL14	TN	Ven + Obi	72	216	25 (12)	At 5 y 65.8/40.6	At 5 y 85.7/60	Al-Sawaf et al. [62,63]
		Clb + Obi	71	216	24 (11)	At 5 y 29.3/15.6	At 5 y 54.2/80.7	
MURANO	R/R	Ven + R	64.5	194	53 (27)	At 5 y 42.5/27.3	At 5 y 88.7/70.2	Seymour et al. [61]
		BR	66	195	55 (28)	Median 19.6/13.4 m	At 5 y 61.8/60.7	
M12-175, M13-365, M13-982, M14-032	R/R	Ven +/− R	66	436	243 (71)	NA	NA	Roberts et al. [59]
VENICE-1	R/R	Ven	68	258	* 35 (14)	* Median 30.5/19.4 m	NA	Kater et al. [60]

Abbreviations: PFS, progression-free survival; OS, overall survival; D, disrupted; ND, not disrupted; NA, not available; Ven, Venetoclax; Obi, Obinutuzuab; Clb, chlorambucil; R, Rituximab; BR, Bendamustine-Rituximab; y, years; m, months; NA, not available. * refers to del(17p) only.

**Table 6:** Efficacy outcomes based on *IGHV* mutational status with BCL2 + BTK inhibitors.

Trial	Setting	Treatment	No. of Patients	Age Median	U-CLL N (%)	PFS% M-CLL/U-CLL	OS% M-CLL/U-CLL	Ref.
CAPTIVATE	TN	Ibr-Ven	195	60	119 (61)	At 3 y 92/88	At 3 y 100/98	Allan et al. [69]
GLOW	TN	Ibr-Ven	106	71	67 (63.2)	At 42 m 90.0/69.8	NA	Niemann et al. [71]
		Clb-Obi	105	71	57 (54.3)	At 42 m 43.1/15.0	NA	
FLAIR	TN	Ibr-Ven	260	62	123 (47.3)	At 3 y 94.3/98.3	At 3 y: 94.3/98.3	Munir et al. [72]
		FCR	263	62	138 (52.5)	At 3 y 88.6/71.2	At 3 y; 88.6/71.2	
CLL13	TN	Ibr-Ven-Obi	231	60	123 (53.2)	At 3 y 96/86.6	NA	Eichhorst et al. [73]
		Ven + R	237	62	134 (56.5	87/76.4	NA	
		Ven + Obi	229	62	130 (57)	82.9/96.6	NA	
		CIT	229	61	131 (57.2)	89.9/65.5	NA	

Abbreviations: PFS, progression-free survival; OS, overall survival; NA, not available; Ibr, ibrutinib; Ven, Venetoclax; Obi, obinutuzuab; Clb, chlorambucil; FCR, fludarabine–cyclophosphamide–rituximab; CIT, Chemoimmunotherapy; y, years; m, months.

**Table 7:** Ongoing clinical trials evaluating MRD as primary outcome.

Trial	Phase	Setting	Treatment	Primary Endpoint
NCT03766763	2	HR TN	Venetoclax	MRD < 0.01% in BM at 12 months
NCT05069051	2	RR	Belimumab + Rituximab-Venetoclax	MRD negativity at the end of treatment
NCT05943496	1b	TN	Tafasitamab + Acalabrutinib + Obinotuzumab	MRD evaluation every 3 months up to 2 years
NCT05197192	3	HR TN	Acalabrutinib + Obinotuzumab-Venetoclax	MRD in BM and PB at the end of study
NCT04908228	2	TN	Ibrutinib + Obinotuzumab	MRD in BM at 30 days therapy initiation
NCT03128879	2	TN	Ibrutinib + Venetoclax Acalabrutinib + Venetoclax	MRD in BM after 12 cycles

Legend: HR, High Risk; TN, Treatment-naive; RR, Relapsed Refractory; MRD, Measurable Residual Disease; BM, Bone Marrow; PB, Peripheral Blood.
